# Correlation of Thyroid Fine Needle Aspiration Biopsy With Histopathological Results

**DOI:** 10.7759/cureus.39130

**Published:** 2023-05-17

**Authors:** Cemalettin Durgun

**Affiliations:** 1 General Surgery, Memorial Dicle Hospital, Diyarbakır, TUR

**Keywords:** thyroid histopathology, thyroid cytopathology, thyroid biopsy, fine needle aspiration, bethesda

## Abstract

Introduction

Fine needle aspiration biopsy (FNAB) is an effective method used in the differential diagnosis of thyroid nodules. The Bethesda system has contributed to the determination of clinical approaches by bringing standardization to cytopathology reporting. However, the rate of cytological-histological incompatibility varies between 10% and 30%. Results differ according to clinics in the literature. These results create a need to reevaluate the efficacy and safety of fine needle aspiration biopsy. In this study, we aimed to evaluate the diagnostic accuracy of FNAB of thyroid nodules by correlating the cytopathology results of FNAB with the results of postoperative histopathology.

Methods

In this retrospective study, thyroid FNAB results and postoperative histopathology results of patients who underwent thyroidectomy operations in our clinic between January 2018 and December 2021 were compared. Accuracy, sensitivity (Sn), specificity (Sp), positive predictive value (PPV), negative predictive value (NPV), false positive rate (FPR), and false negative rate (FNR) were calculated. Cases with nondiagnostic FNAB results were excluded from the calculations. FNAB results with a follicular neoplasm/suspicious for a follicular neoplasm (FN/SFN) and suspicious for malignancy were included in the malignant group.

Results

A total of 304 patients were included in the study. The male/female ratio was 1:3.3. As a result of the study, malignancy was detected histopathologically in 47 (15.46%) patients. The commonest malignancy detected was papillary carcinoma. According to the Bethesda system, the results were evaluated in six categories. The incidence of malignancy in the Bethesda categories were 0%, 4%, 40%, 69.2%, 100%, and 100%, respectively. Accordingly, the specificity and sensitivity of FNAB for detecting malignancy were 98.7% and 66.6%, respectively. The accuracy was 93.5%. The false positive rate, false negative rate, positive predictive value, and negative predictive value were 1.20%, 33.3%, 91.4%, and 93.8%, respectively.

Conclusion

Thyroid FNAB is an effective method used with satisfactory reliability in the differential diagnosis of malignancies of thyroid nodules. Still, it has some limitations. This article demonstrates higher rates of malignancy in Bethesda categories III and IV. Therefore, clinical approaches are gaining importance in these categories.

## Introduction

Thyroid nodules are one of the most common endocrine pathologies in the community. Its incidence in autopsy series is up to 65% [1]. Its prevalence is even higher in iodine-deficient countries. They are detected at a rate of 19%-35% in clinical and ultrasound examinations [2,3]. In general, the malignancy rate of these nodules is around 7%-15% [4]. Thyroid cancers are the most common endocrine cancer [5]. The incidence of thyroid cancer worldwide has recently increased significantly compared to other cancers [5,6]. The main goal in the clinical management of thyroid lesions is to make the differential diagnosis of malignant lesions. Fine needle aspiration biopsy (FNAB) is the most common and effective method used for this purpose. Thyroid FNAB is a minimally invasive method that is well tolerated by patients with a low complication rate [7].

FNAB has a very important place in the decision of surgical and conservative treatment. By identifying nodules suspected of malignancy, it provides early diagnosis and surgery and also prevents unnecessary surgical procedures. In clinical management, surgical procedures ranging from lobectomy to total thyroidectomy and even central neck dissection are determined according to FNAB results. However, the procedure has certain limitations due to false positive (FP) and false negative (FN) results. The rate of cytological and histological incompatibility varies between 10% and 30%. Results differ between clinics [8,9]. This situation creates the need to reevaluate the efficacy and safety of FNAB. In this study, we aimed to discuss the efficacy and accuracy of FNAB by correlating FNAB cytopathology results with postoperative histopathology results.

## Materials and methods

In this study, the files of patients who underwent thyroidectomy at Memorial Dicle Hospital between January 2018 and December 2021 were reviewed retrospectively. Demographic characteristics of the patients, thyroid FNAB results, and postoperative final pathology results were recorded.

Patients whose thyroid FNAB results could not be obtained or who underwent surgery without the need for FNAB or whose histopathology results could not be obtained were excluded from the study. The histopathology results of the patients included in the study were correlated with the preoperative FNAB cytopathology results. The study was approved by the Ethics Committee of Memorial Şişli Hospital with the decision dated 12.11.2021/007. Informed consent was obtained from all patients.

FNABs were performed in all patients by interventional radiology or endocrinology units, accompanied by ultrasonography. The decision for surgery was made based on the combined findings of the FNAB and clinical examination. Histopathological and thyroid FNAB evaluations of the patients were performed in the pathology laboratories of our hospital.

In patients with multiple FNAB results or multiple nodules, if one of the results included suspicion of malignancy, the patient was evaluated in the suspicious for malignancy group. FNAB results were classified according to the 2017 Bethesda system [10]. The Bethesda system was updated in 2017, and patient management recommendations accordingly are summarized in Table 1.

**Table 1:** Bethesda System for Reporting Thyroid Cytopathology and recommended clinical management FNAB: fine needle aspiration biopsy

Diagnostic category	Description	Management
I	Nondiagnostic or unsatisfactory	Repeat FNAB with ultrasound guidance
II	Benign	Clinical and sonographic follow-up
III	Atypia of undetermined significance or follicular lesion of undetermined significance	Repeat FNAB, molecular testing, or lobectomy
IV	Follicular neoplasm or suspicious for a follicular neoplasm	Molecular testing, lobectomy
V	Suspicious for malignancy	Near-total thyroidectomy or lobectomy
VI	Malignant	Near-total thyroidectomy or lobectomy

In statistical analysis, accuracy, sensitivity (Sn), specificity (Sp), positive predictive value (PPV), negative predictive value (NPV), false positive rate (FPR), and false negative rate (FNR) were calculated. Cases with nondiagnostic/unsatisfactory FNAB results were excluded from the calculations. FNAB results with a follicular neoplasm/suspicious for a follicular neoplasm (FN/SFN) and suspicious for malignancy were included in the malignant group. Descriptive statistics of the data were calculated as percentages and numbers.

## Results

A total of 304 patients were included in this study. Of the patients, 234 (76.98%) were female. The median age of the patients was 45.9 (range: 15-87). The female-to-male ratio was found to be 3.3:1. As a result of the study, malignancy was detected histopathologically in 47 (15.46%) patients. The most common malignant lesions were papillary carcinoma (n = 33, 70.21%). Follicular carcinoma took second place (n = 12, 25.53%).

In the FNAB results evaluated according to the Bethesda classification, 10 (3.29%) patients were reported as nondiagnostic/insufficient material, 244 (80.26%) as benign cytology, 15 (4.93%) as atypia/follicular lesion of undetermined significance (AUS/FLUS), 13 (4.27%) as FN/SFN, 12 (3.95%) as suspicious for malignancy, and 10 (3.29%) as malignant.

Hashimoto’s thyroiditis was found in one patient, and nodular colloidal goiter was found in nine patients in the histopathology results of the patients whose FNAB results were nondiagnostic. Of the 22 patients diagnosed with Hashimoto’s thyroiditis, one was reported as nondiagnostic cytology, three as AUS/FLUS, and 18 as benign cytology in the Bethesda system.

In the histopathology results of 13 patients whose FNAB results were FN/SFN, follicular carcinoma was detected in eight patients, papillary carcinoma in one, nodular colloidal goiter in two, follicular adenoma in one, and Hürthle cell adenoma in one. In this category, the malignancy rate was found to be 69%.

In the histopathology of patients with AUS/FLUS as a result of FNAB, three were reported as Hashimoto’s thyroiditis, four as nodular colloidal goiter, two as follicular adenoma, four as papillary carcinoma, one as follicular carcinoma, and one as medullary carcinoma. The malignancy rate was 40%. The malignancy rates found at the end of the study and the malignancy rates reported in the Bethesda system are shown in Table 2.

**Table 2:** Implied risk of malignancy per each category of the Bethesda system compared with malignancy rates of our study

Bethesda	Risks of malignancy (% )	Result (%)
I	5-10	0
II	0-3	4
III	~10-30	40
IV	25-40	69.2
V	50-75	100
VI	97-99	100

In our study, thyroid carcinoma was detected in all patients whose FNAB results were malignant and suspicious for malignancy. Of the patients with suspected malignancy as a result of FNAB, 10 were papillary carcinoma and two were follicular carcinoma. One patient resulted in medullary thyroid carcinoma, and nine patients had papillary carcinoma, which was reported as malignant cytology in the Bethesda VI category. All eight cases reported as papillary carcinoma as a result of FNAB were also reported as papillary carcinoma in histopathology. The findings are summarized in Table 3.

**Table 3:** Comparison of fine needle aspiration cytopathology with histopathology DC: diagnostic category

Bethesda DC	Number (%)	Benign (number (%))	Malignant (number (%))	Type of malignancy (number (%))		
				Papillary carcinoma	Follicular carcinoma	Medullary carcinoma
I	10 (3.29)	10 (100)	0 (0)	0 (0)	0 (0)	0 (0)
II	244 (80.26)	234 (96)	10 (4)	9 (90)	1 (10)	0 (0)
III	15 (4.93)	9 (60)	6 (40)	4 (67)	1 (16)	1 (16)
IV	13 (4.27)	4 (30)	9 (70)	1 (11)	8 (89)	0 (0)
V	12 (3.95)	0 (0)	12 (100)	10 (83)	2 (17)	0 (0)
VI	10 (3.29)	0 (0)	10 (100)	9 (90)	0 (0)	1 (10)

In the statistical analysis, patients whose FNAB results were considered nondiagnostic/insufficient material were excluded from the calculations. FNAB results with an FN/SFN and suspicious for malignancy were included in the malignant group. The calculations were performed as follows: Sn = TP/(TP + FN) = 32/(32 + 16) = 66.6%, Sp = TN/(TN + FP) = 243/(243 + 3) = 98.7%, PPV = TP/(TP + FP) = 32/(32 + 3) = 91.4%, NPV = TN/(TN + FN) =243/(243 + 16) = 93.8%, FPR = FP/(FP + TN) = 3/(3 + 243) = 1.2%, FNR = FN/(FN + TP) = 16/(16 + 32) = 33.3%, accuracy = TP + TN/total number of cases = 32 + 243/294 = 93.5%.

The Sp and Sn of FNAB for detecting malignancy were 98.7% and 66.6%, respectively. FPR, FNR, PPV, and NPV were 1.20%, 33.3%, 91.4%, and 93.8%, respectively. The accuracy of FNAB was 93.5%.

## Discussion

Thyroid FNAB is a low-cost, minimally invasive method with low complication rates used in the differential diagnosis of thyroid nodules [11]. With the Bethesda System for Reporting Thyroid Cytopathology, standardization was achieved in FNAB classification, and patient management was facilitated by reducing diagnostic inconsistencies. According to this classification, the results were evaluated in six categories, and predicted malignancy rates were reported.

Thyroid cancer is a common cancer in pathology specimens because it does not cause symptoms in the early stages. In our study, the malignancy rate was 15.46%. In general, the malignancy detection rate in FNAB varies between 5% and 10%, while this rate is reported as 2.6%-10.7% in surgical materials [12]. The malignancy rates we found seem to be slightly higher than those in the literature. This is due to the fact that most patients who were reported as benign, as a result of FNAB, were followed closely without surgery. This is proof that FNAB reduces the number of unnecessary surgeries. There are also studies in the literature reporting similar results to ours [13,14].

The reason for the gradual increase in thyroid cancer is due to the increased incidence of papillary thyroid carcinoma. In our study, 70.21% of malignant cases were papillary carcinoma. This was followed by follicular carcinoma and then medullary carcinoma. The findings were consistent with the literature [15].

In this study, malignancy was detected in 69.2% of the cases reported as FN/SFN. This rate is higher than the predicted malignancy rate in the Bethesda system (25%-40%). However, there are studies reporting a malignancy rate of 50%-79% in Bethesda category IV [16]. These results reveal that patients with FN/SFN can also be operated on with a malignancy-like approach.

We detected malignancy in 40% of patients whose FNAB results were reported as AUS/FLUS. This rate is higher than the risk of malignancy defined in Bethesda 2017. The data indicate a higher incidence of malignancy in this class. Similarly, there are studies in the literature reporting a malignancy rate of 35%-53% in this class [9,17,18]. These results require a repeat biopsy or lobectomy as recommended in the Bethesda system.

Thyroid FNAB leads to a decrease in the number of unnecessary surgeries but also increases the rate of malignancy reported as a result of histopathology [19]. The detection of malignancy in the histopathology results of all patients whose FNAB results were malignant or suspicious for malignancy was a remarkable success in our study. This rate is reported as 99%-97% in the Bethesda system. In the study of Zarif et al. [20], 100% of the cases diagnosed with malignant cytology were diagnosed as malignant.

As a result of our study, the sensitivity and specificity that we found were compatible with the literature [21]. In the literature, FNAB sensitivity rates vary between 65% and 98%, with a specificity of 72%-100% [22]. The reason for these differences depends on the experience of the operator performing the procedure, the use of ultrasound-guided biopsy technique, and the classification of suspicious lesions [23,24]. As a result of our study, the accuracy, false positive rate, false negative rate, positive predictive value, and negative predictive value of thyroid FNAB for detecting malignancy were consistent with the literature [25]. The results obtained in the statistical analysis show that FNAB has a moderately satisfactory ability to detect thyroid malignancies.

This study has some limitations due to its retrospective nature. The patients included in the study consisted of those who had both FNAB results and underwent surgery. Therefore, the histopathology results of patients who did not undergo surgery are unknown. Also, due to the nature of this retrospective study, it is limited to regional outcomes.

## Conclusions

FNAB is an effective method for the differential diagnosis of thyroid nodules, providing a satisfactory level of reliability. This particular article emphasizes that thyroid nodules classified under Bethesda categories III and IV exhibit higher rates of malignancy.

In the classification made according to the Bethesda system, the results in category III should be approached cautiously and the biopsy should be repeated. The malignancy rates we encountered in the Bethesda category IV are at a substantial level. Therefore, the clinical approach for this category needs to be examined as in the malignant group.
